# Attempt to Make the Upper-Limb Item of Objective Fugl–Meyer Assessment Using 9-Axis Motion Sensors

**DOI:** 10.3390/s23115213

**Published:** 2023-05-30

**Authors:** Yusuke Ueyama, Takashi Takebayashi, Kenta Takeuchi, Makoto Yamazaki, Keisuke Hanada, Yuho Okita, Shinichi Shimada

**Affiliations:** 1 Department of Occupational Therapy and Rehabilitation, Itami Kousei Neurosurgical Hospital, Itami City 664-0028, Japan; 2 Department of Occupational Therapy, School of Comprehensive Rehabilitation, Osaka Metropolitan University, Habikino City 558-8555, Japan; takshi77@omu.ac.jp; 3 Department of Physical Medicine and Rehabilitation, Itami Kousei Neurosurgical Hospital, Itami City 664-0028, Japan; 4 Faculty of Rehabilitation, Shijonawate Gakuen University, Daitou City 574-0011, Japan; 5 Faculty of Health, Arts and Design, Swinburne University of Technology, Melbourne, VIC 3122, Australia; 6 Department of Neurosurgery, Itami Kousei Neurosurgical Hospital, Itami City 664-0028, Japan

**Keywords:** Fugl–Meyer Assessment, upper limb, VICON, IMU

## Abstract

The Fugl–Meyer Assessment (FMA) has been used as a functional assessment of upper-limb function in stroke patients. This study aimed to create a more objective and standardized evaluation based on an FMA of the upper-limb items. A total of 30 first-ever stroke patients (65.3 ± 10.3 years old) and 15 healthy participants (35.4 ± 13.4 years old) admitted to Itami Kousei Neurosurgical Hospital were included. A nine-axis motion sensor was attached to the participants, and the joint angles of 17 upper-limb items (excluding fingers) and 23 FMA upper-limb items (excluding reflexes and fingers) were measured. From the measurement results, we analyzed the time-series data of each movement and obtained the correlation between the joint angles of each part. Discriminant analysis showed that 17 and 6 items had a concordance rate of ≥80% (80.0~95.6%) and <80% (64.4~75.6%), respectively. In the multiple regression analysis of continuous variables of FMA, a good regression model was obtained to predict the FMA with three to five joint angles. The discriminant analysis for 17 evaluation items suggests the possibility of roughly calculating FMA scores from joint angles.

## 1. Introduction

According to the American Heart Association/American Stroke Association Guidelines, it is necessary to incorporate standardized, validated, and formal measures for the rehabilitation care of adults recovering from stroke [1]. The Fugl–Meyer Assessment (FMA) was developed by Fugl–Meyer et al. [2] in 1975. The significance of implementing an FMA for the affected upper extremity after stroke was ensured by a three-step selection strategy developed by Baker et al. [3]. The motor tasks of the FMA were designed based on the fact that the hemiplegic recovery process after a stroke can be gradual so that the recovery stages are defined by the orders [2,4]. The reliability, validity, and responsiveness of this assessment have been ensured through many classical test theories [5,6,7,8,9,10,11]. Recent studies on upper-limb outcome measures have shown that the FMA is the most commonly used outcome measure in stroke rehabilitation [12]. A previous study by Sullivan et al. suggested the FMA’s significantly high inter-rater reliability score of 0.98 [13]. However, FMA scores are not always the same, and errors can occur when examiners without FMA administration training evaluate stroke patients because the FMA is an examiner’s subjective assessment of their physical and motor abilities. In addition, it is challenging to capture precise changes in each movement, and the scores may differ even if the same examiner evaluates the same movement. Therefore, administrative training to perform an FMA is needed to reduce the score gap.

There are also several concerns in implementing an FMA, including the large number of test items (33) and the time and effort required to evaluate each task [7,11].

To address these issues, kinematic evaluations using robotic devices have been implemented more widely (since 2010). More specifically, a higher number of reports explain the high usability in upper-limb evaluation with the use of human-mounted multi-axis sensors, glove-type sensors, and Kinect, which contain acceleration and velocity sensors [14,15,16,17,18,19].

Optical motion capture systems have been used for motion analyses in biomechanics. A reflective marker was attached to the body, and a high-resolution camera was used to track the position of the marker and measure posture and joint angles. VICON (Oxford, UK) is an optical motion capture system that has become the gold standard for motion analysis [20].

However, VICON has some disadvantages, such as limited space and high cost.

Kinect (Microsoft, New York, NY, USA), like VICON, is used for motion analysis in biomechanics; it is less expensive and easier to implement than VICON and has been used in many recent motion analyses. However, it has some limitations, such as the occlusion of body parts during tracking using Kinect. In addition, it cannot track the forearm pronation/supination, radial flexion/ulnar flexion, joints beyond the wrist joint, or whole-body tracking [17].

When validating automated FMA, machine learning algorithms (random forests [14], machine learning [15], support vector machines [16], artificial neural networks [17]) are now seen to be used to classify from the acquired data. Although these methods have the potential for high predictive performance, they are difficult to implement in practice because of the large amount of data required for training and the need to reduce data bias, which requires a wide variety of data.

The inertial measurement unit (IMU), which consists of accelerometers, gyroscopes, and magnetometers to measure the orientation and velocity of objects, is recognized as a means of overcoming the shortcomings of existing optical motion capture systems.

The results of measuring the range of motion of joints with VICON and IMU, and VICON and Kinect, showed high correlation, and the accuracy was comparable to that of existing optical sensors [21,22].

This study analyzed the relationship between the values measured by the sensor and the FMA scores by implementing the participants wearing a nine-axis motion sensor when evaluating the FMA of the upper-limb items. Furthermore, this study examined whether a standardized algorithm for the FMA of upper-limb items could be created. Finally, the purpose of this study was to create a more objective and standardized evaluation based on the FMA of the upper-limb items.

## 2. Materials and Methods

### 2.1. Research Design

This is a single-center cross-sectional observational study.

### 2.2. Participants

All participants were recruited between September 2017 and March 2018 at the Itami Kousei Neurosurgery Hospital in Japan. We recruited 30 patients with a first-ever stroke and 15 healthy participants within the age group of 20–80 years who consented to participate in the study. The background information of the participants was collected from their electronic medical records. Additionally, the principal investigator (Y.U.) of this study conducted an interview to collect further information. We also collected the following information: age, sex, dominant hand (right/left), date of onset, type of stroke, upper limb, Brunnstrom stage of the hand (hereafter referred to as upper limb Br. stage and hand Br. stage), and sensory impairment.

The inclusion criteria for the stroke patient group were as follows: (1) motor paralysis of the unilateral upper limb due to a first-ever stroke, (2) hospitalization at the Itami Kousei Neurosurgery Hospital for rehabilitation purposes, and (3) ≥20 years at the time of consent. The inclusion criteria for the normal participants were as follows: (1) no impairments and (2) those aged ≥ 20 years at the time of consent. Exclusion criteria were as follows: (1) no capacity to sit independently; (2) severe cognitive impairment; (3) no capacity to assume the limb position during calibration of the motion sensor; (4) inability to wear one of the motion sensors on the upper spine, pelvis, or upper limbs because of skin diseases; (5) bilateral motor paralysis; (6) inability to perform the evaluation due to the risk of stroke or other diseases decided by the physician or the therapist; and (7) severe upper limb impairment due to stroke unrelated medical reasons. To confirm the exclusion criteria, the principal investigator (Y.U.) interviewed patients through electronic medical records and rehabilitation staff.

### 2.3. Ethical Considerations

This study was approved by the Ethics Review Committee of MINS, a nonprofit organization, as well as the Ethics Committee of Osaka Prefecture University (approval number: 2020-211).

### 2.4. Measurement Equipment

**Figure 1:**
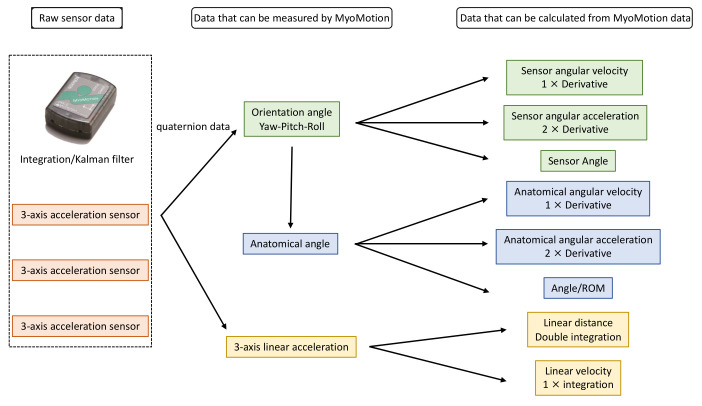
Principle of MyoMotion measurement.

**Figure 2:**
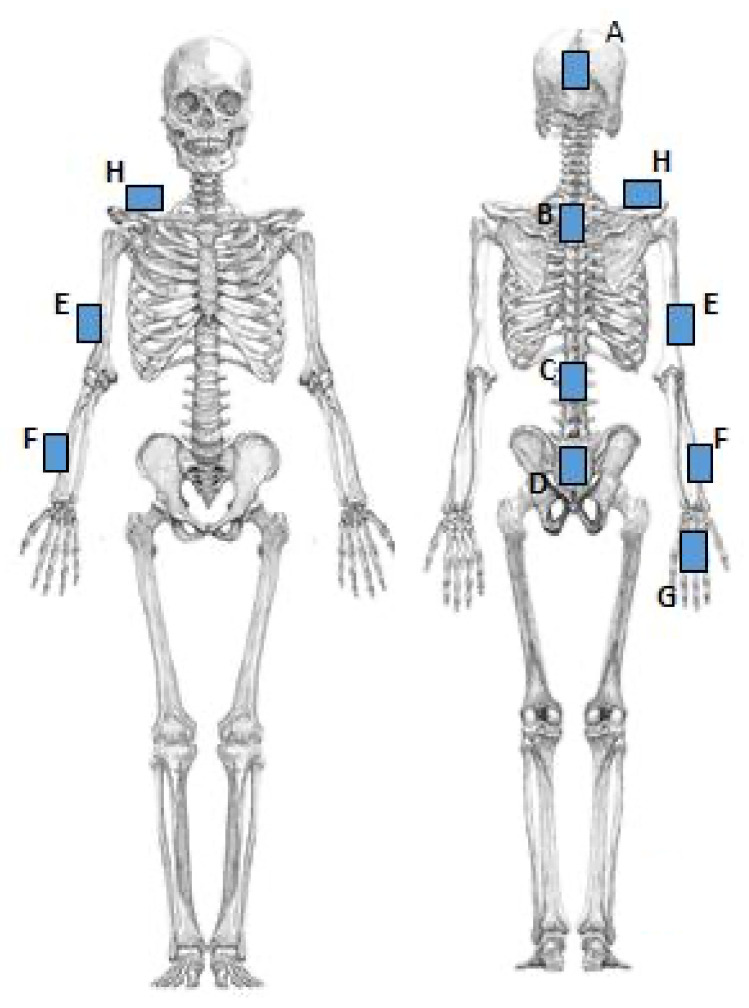
The installation location of the motion sensor. A: Head. B: C7. C: Th12. D: Pelvis. E: Upper arm. F: Forearm. G: Back of the hand. H: Upper trapezius.

Noraxon MyoMotion (Scottsdale, AZ, USA) was used to analyze motion variables. A small inertial measurement unit (IMU) installed on each body segment tracked the 3D angular orientation. IMU sensors include a 3-axis accelerometer, a 3-axis gyroscope, and a 3-axis magnetometer. These data were integrated using a fusion algorithm to obtain the quaternion data for each sensor. Each parameter was calculated from quaternion, linear acceleration, and magnetometer data (Figure 1). The IMU sensor was completely wireless and transmitted the measurement data to the wireless receiver of the MyoMotion system MR3.10 (Noraxon), thus eliminating the need for calibration of the measurement space. The IMU sensor can measure angles with a static accuracy of ±0.4° and a dynamic accuracy of ±1.2°. In this evaluation, sensors were attached to the hand occiput, head, C7, Th12, pelvis, upper trapezius fibers, upper arm, forearm, and back of the hand (Figure 2).

### 2.5. Experimental Procedure

**Figure 3:**
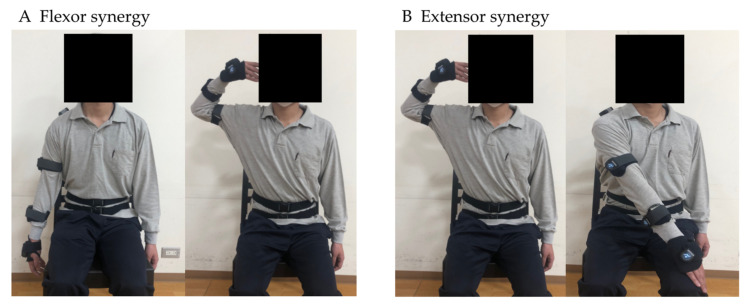
Example of FMA upper-extremity items.

All participants were examined by the principal investigator, who also worked as an occupational therapist. Participants sat in a chair by themselves or with the examiner’s help, and the test items were (1) passive range of motion (P-ROM) and (2) FMA upper-extremity items. As first, the IMU sensor was calibrated, and the passive range of motion was measured. Calibration was performed in a seated position in a chair with the upper extremity straight down along the body. P-ROM assessments were performed in the same limb position as upper-extremity FMA items to determine if range of motion was limited in the following movements: (1) shoulder flexion, extension, and abduction with elbow extension; (2) shoulder internal/external rotation, shoulder abduction, and elbow flexion at 90°; (3) elbow flexion/extension; (4) forearm internal/external rotation, 90° elbow flexion, and mid-forearm position; (5) hand palmar flexion and dorsiflexion: 90° elbow flexion and forearm rotation; (6) forearm pronation and supination: 30° shoulder joint flexion and abduction, elbow joint extension, and mid-forearm position; (7) palm flexion/dorsiflexion of the wrist: 30° shoulder flexion/abduction, elbow extension, and forearm rotation; and (8) hand radial/ulnar flexion, 90° elbow flexion, and forearm rotation. After P-ROM assessment, the IMU sensor was calibrated again, and the upper-extremity items on the FMA were measured. For the upper-extremity FMA items, the examiner was instructed verbally and by imitation on how to perform the movements (Figure 3). The movement method was evaluated according to the description by Nagata [23]. However, the item “C. Hand” was excluded from this study because measuring the data with a motion sensor was difficult (Table 1).

**Figure 4:**
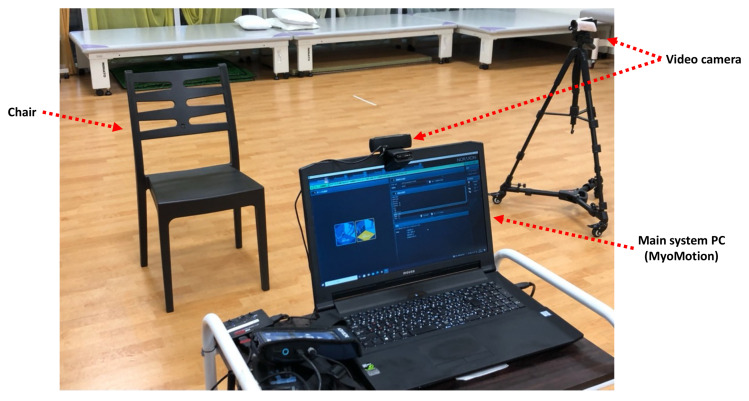
Measurement environment and video camera location.

In addition, video recordings were conducted using two cameras from the front and the affected side to confirm the movement (Figure 4).

For each FMA task, we marked the starting limb position, the final limb positions that the participants reached spontaneously, and the ending limb position with a manual switch linked to MyoMotion’s PC during data extraction.

### 2.6. Extraction of Data

The following data were obtained: 17 items of the joint angle, time-series data of each FMA movement, and joint angle of the final limb position. For the joint angles, we used the joint angle data of the anatomical angles that could be measured using the IMU sensor. Anatomical angles were calculated from two adjacent sensors with a calibration state of 0° (e.g., shoulder abduction was calculated from the upper trapezius fibers and upper arm IMU sensors. The joint angle data were as follows: (1) neck flexion/extension, (2) neck lateral flexion (left/right), (3) neck rotation (left/right), (4) chest flexion/extension, (5) chest lateral flexion (left/right), (6) chest rotation (left/right), (7) waist flexion/extension, (8) waist lateral flexion (left/right), (9) waist rotation (left/right), (10) shoulder joint flexion/extension, (11) shoulder joint abduction/adduction, (12) shoulder joint internal/external rotation, (13) shoulder joint total flexion, (14) elbow joint flexion/extension, (15) forearm pronation/supination, (16) wrist palmar flexion/dorsiflexion, and (17) wrist radial flexion/ulnar flexion (Table 2).

### 2.7. Analysis Method

Multivariate analysis was conducted for the obtained movement analysis to evaluate the quantitative characteristics of the movements and to analyze the relationship with the results of the FMA evaluation of each participant in the following order: (1) analyze the time-series data of each movement and obtain the correlation between the joint angles of each part; (2) perform multiple regression analysis using the FMA evaluation items of flexor synergy, extensor synergy, and coordination and speed as objective variables and joint angles of the final limb position of each movement as explanatory variables; (3) construct a discriminant equation using the joint angle of the final limb position, and evaluate the misjudgment rate.

## 3. Results

### 3.1. Participants

In total, 45 participants (30 patients with first-ever stroke and 15 healthy participants) were included in this study. The attribute data of the participants are presented in Table 3. None of the participants had limitations in the range of motion in the P-ROM.

### 3.2. Analysis of Time Series Data of Movements

**Figure 5:**
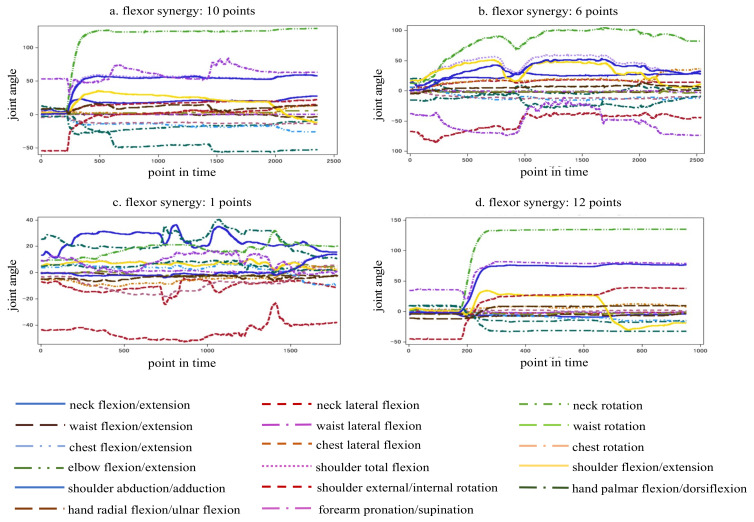
Sample analysis of time-series movement data.

Figure 5 indicates the time-series data of joint angles from the start to the end during flexor synergy movement. Figure 5a–c show the data samples of the stroke patients and Figure 5d shows those of a healthy participant. The vertical axis represents the joint angle, and the horizontal axis represents time. The time was measured at 0.01 s intervals, with 1000 points corresponding to 10 s. With data from a single point, blurring of the line and compensatory motion can be observed, and the line is not smooth because the motion does not stop. As the number of points increases, the compensatory motion decreases; thus, the line gradually becomes less blurred. With 12 data points, the compensatory motion was not observed, and the line was smooth because the motion could be stopped.

### 3.3. Correlation between Joint Angles of Each Part

The results of the correlation coefficients for flexor synergy scores are shown (Supplementary Tables S1–S3). Pearson’s correlation coefficient showed that the higher FMA score, the higher the correlation coefficient between each trunk movement (flexion, lateral flexion, rotation) and upper-extremity joint angles in near-full items. In patients with lower FMA scores (i.e., flexor synergy 1 point; Supplementary Table S3), the correlation coefficients were higher for trunk flexion–shoulder flexion/extension, abduction/adduction, elbow flexion/extension, wrist radial flexion/extension, trunk lateral flexion–shoulder abduction/adduction, and trunk rotation–forearm internal/external rotation.

### 3.4. Relationship between FMA (Continuous Variable) and Joint Angle

First, multiple regression analysis was conducted with (1) flexor synergy (0–12 points), (2) extensor synergy (0–6 points), and (3) coordination and speed (0–6 points) as objective variables, and joint angle data (17 items) of the final limb position of each movement as explanatory variables. The following results were obtained: (1) elbow flexion/extension, shoulder flexion/extension, shoulder internal/external rotation (R2 = 0.823) in flexor synergy; (2) elbow flexion/extension, neck rotation, shoulder total flexion, shoulder flexion/extension, shoulder internal/external rotation (R2 = 0.734) in extensor synergy; (3) elbow flexion/extension, shoulder total flexion, shoulder internal/external rotation (R2 = 0.721) in coordination and speed (Table 4).

Multiple regression analysis was performed using each FMA (continuous variable), and one variable of the most relevant final limb position was used as the explanatory variable.

**Figure 6:**
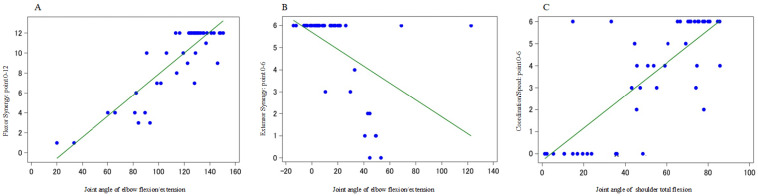
Multiple regression analysis with one variable of the final limb position most associated with each endpoint of the FMA as an explanatory variable. (**A**): Joint angle of “elbow flexion/extension” of the FMA assessment value and the final limb position during flexor synergy. (**B**): Joint angle of “elbow flexion/extension” of the FMA assessment value and the final limb position during extensor synergy. (**C**): Joint angle of “shoulder total flexion” of the FMA assessment value and final limb position during coordination/speed.

The following results were obtained: (1) flexor synergy = −2.65 + 0.106 × elbow flexion/extension (R2 = 0.763), (2) extensor synergy = 5.70 − 0.038 × elbow flexion/extension (R2 = 0.261), and (3) coordination/speed = −0.319 + 0.074 × shoulder total flexion (R2 = 0.599). These results indicate that the joint angle of the elbow flexion/extension can be estimated based on the FMA score during flexor joint exercises. However, the prediction accuracy of the FMA scores for the joint exercises of the extensor muscles appeared to be poor using only the joint angle of elbow flexion/extension, and for the coordination/speed, the FMA score fluctuated greatly when the shoulder total reflection was approximately 30–50°; therefore, it was necessary to combine other explanatory variables in both cases (Figure 6).

### 3.5. Construction of Discriminant Equation and Misjudgment Rate Using a Joint Angle of Final Limb Position

**Figure 7:**
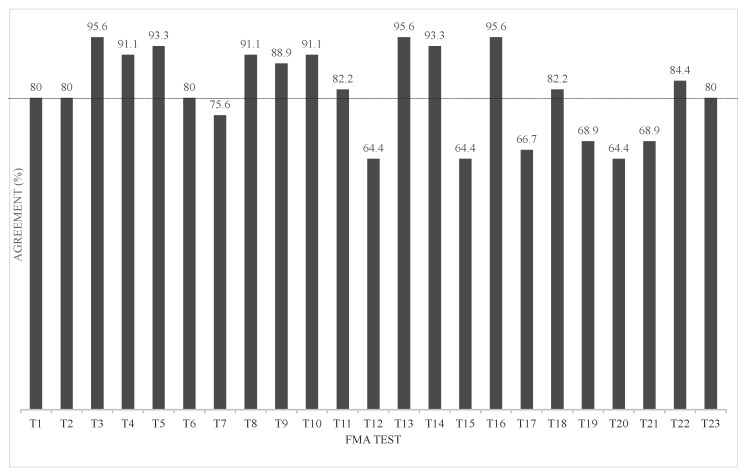
Discriminant analysis classification by agreement rate.

We conducted a discriminant analysis using the FMA scores (0, 1, 2) as the objective variables and the joint angle data of the final limb position of each movement (17 items) as the explanatory variables. These were used to evaluate misclassification rates. The results showed that the agreement rate was 80% and more (80.0–95.6%) for 16 items; however, the rest of the seven items showed an agreement rate below 80% (64.4–75.6%). In particular, the discrepancy rate was larger for movements including “forearm rotation/external rotation” and “palmar dorsiflexion” (Figure 7). Surprisingly, there were 12 out of 23 FMA items in which several chest or waist movements were chosen as the variable (Supplementary Table S4).

## 4. Discussion

The reason for the larger discrepancy rate in the movements including “forearm adduction/external rotation” and “palmar dorsiflexion” is thought to be the influence of compensatory movements such as shoulder adduction/abduction and trunk lateral flexion during forearm adduction/external rotation and shoulder flexion/extension and elbow flexion/extension during palmar dorsiflexion. However, the motion sensor predicted the score regardless of the presence of compensatory movements, which explains the discrepancy between the examinee’s FMA score and the motion sensor’s score, resulting in a reduced agreement rate.

A study conducted by Kim et al. [17] investigated 13 FMA items using Kinect, looking at the agreement rate between the FMA ratings of occupational therapists and the data obtained using principal component analysis and artificial neural network learning. The results showed that the overall agreement rate was 65–84%. The nine items (including shoulder abduction, external rotation, and elbow flexion) exceeded 70% agreement. However, the following items were performed at an agreement rate of 60–70%: forearm internal/external rotation, hand to lumbar, shoulder abduction 90°, and shoulder flexion 180°.

The study by Formstone et al. [19] investigated 12 FMA items, excluding reflexes and hand items. This study used accelerometers attached to the upper arm and forearm and examined the agreement between the clinician’s FMA ratings and the linear support vector machine. The results showed a low accuracy of 62.0% (44.3–74.3%).

Multiple regression analysis for continuous variables of the FMA showed a good regression model that predicted the FMA for three to five joint angles. Furthermore, discriminant analysis was used for the FMA evaluation points (0, 1, and 2) of the single movements. The explanatory variables were selected, and a linear discriminant function was constructed to examine the predictability of the FMA to which the participant belonged. The agreement rate was >80% and <80% for 17 and 7 items, respectively.

As mentioned earlier, this study aimed to objectively examine the FMA by attaching motion sensors to eight locations. The results suggested that 17 of 33 FMA upper-extremity items could be measured accurately using three to five joint angles to predict FMA scores, which appeared to indicate better outcomes than those of previous studies. In previous studies [19], sensors were attached only to the upper arm and fingers and not to the body trunk. In addition, Kinect-based studies [17] have only used joint data from the upper limbs and head. In this study, we attached motion sensors to the back of the head, C7, Th12, pelvic region, and head and body trunk. There was a high correlation between upper extremity and trunk movements when performing the FMA, and some items in the discriminant analysis used several trunk movements (Supplementary Table S4). The higher prediction accuracy of this study compared to previous studies [17,19] can be attributed, in part, to the selection of the trunk as a variable. Therefore, adding trunk movement as a variable is necessary to improve the prediction accuracy of the FMA.

Yoon [24,25] investigated the reliability and validity of the range of motion of the neck and shoulder joints using MyoMotion. The reliability was relatively good, with intraclass correlation coefficients (ICCs) of (3.2) 0.63–0.98 for the neck and ICCs of (3.2) 0.43–0.98 for the shoulder joint, using the test–retest method. Validity was compared between the range of motion data obtained from the IMU, goniometer, and joint angle data using photographic measurements, and valid results were obtained for both the neck and shoulder joints. Based on these results, we believe that the range of motion data obtained from MyoMotion is reliable and that valid results were obtained in this study.

Although good results were obtained compared with findings of previous studies, there are limitations in objectifying the FMA upper-extremity items with motion sensors alone, such as body parts that seem impossible to measure with motion sensors (e.g., hand items) and the remaining 16 items for which the agreement rate was low. In future studies, it is necessary to verify the characteristics and trends of measurement and compensatory movements using glove-type sensors, such as Leap Motion and Kinect, and to verify whether it is possible to measure evaluation items that have been difficult to measure and to improve their accuracy. Furthermore, it is necessary to develop applications and work toward automating and reducing the time required for the FMA.

## 5. Conclusions

The multiple regression analysis for continuous variables of FMA showed an excellent regression model that predicted FMA with three to five joint angles. Discriminant analysis showed that the agreement rate was greater than 80% for the 17 evaluation items.

These results suggest that it may be possible to calculate a rough FMA score from joint angles. This could reduce the variability of FMA scores and shorten the evaluation time.

The accuracy of the FMA score could be further improved by combining the FMA score with other instruments; therefore, further research is needed.

## Figures and Tables

**Table 1:** Target Fugl–Meyer assessment of the upper extremity.

Upper Extremity				Target Test Symbol
Reflex activity	Flexors	Data		N/A
	Extensors	Data		N/A
II. Volitional movement within synergies	Flexor synergy	Shoulder	Retraction	T1
			Elevation	T2
			Abduction (90°)	T3
			External rotation	T4
		Elbow	Flexion	T5
		Forearm	Supination	T6
	Extensor synergy	Shoulder	Adduction/internal Rotation	T7
		Elbow	Extension	T8
		Forearm	Pronation	T9
III. Volitional movement mixing synergies	Hand to lumbar spine			T10
	Shoulder flexion 0–90°			T11
	Pronation/supination of the forearm, with the elbow flexed to 90°			T12
IV. Volitional movement with little or no synergy	Shoulder abduction 0–90°			T13
	Shoulder flexion 90–180°			T14
	Pronation/supination of the forearm, with the elbow fully extended			T15
V. Normal reflex activity	Biceps, triceps, finger flexors			N/A
B. **Wrist**	Wrist stability at 15° dorsiflexion with the elbow flexed at 90°			T16
	Repeated wrist flexion and extension with the elbow flexed to 90°			T17
	Wrist stability at 15° dorsiflexion with the elbow at 0°			T18
	Repeated wrist flexion and extension with the elbow at 0°			T19
	Circumduction of the wrist			T20
C. **Hand**	Mass flexion			N/A
	Mass extension			N/A
	Grasp A: extension of the MCP, flexion of the PIP and DIP			N/A
	Grasp B: extended index finger and thumb			N/A
	Grasp C: pulp of the thumb against the pulp of the index finger			N/A
	Grasp D: volar surface on the thumb and index finger against each other			N/A
	Grasp E: spherical grasp			N/A
D. **Coordination/Speed**	Tremor			T21
	Dysmetria			T22
	Time			T23

Note: N/A, not applicable.

**Table 2:** Joint angle data.

Articular Point	Joint Angle
Neck	Flexion/extension
	Lateral flexion (right/left)
	Rotation (right/left)
Chest	Flexion/extension
	Lateral flexion (right/left)
	Rotation (right/left)
Waist	Flexion/extension
	Lateral flexion (right/left)
	Rotation (right/left)
Shoulder	Flexion/extension
	Abduction/adduction
	External rotation/internal rotation
	Total flexion
Elbow	Flexion/extension
Forearm	Pronation/supination
Hand	Palmar flexion/dorsiflexion
	Radial flexion/ulnar flexion

Note: Total flexion, the angle between the trunk and upper limb. Trunk movement was defined as the sum of chest and waist movements (i.e., the sum of chest and waist flexion indicates trunk flexion, and the sum of chest and waist rotation indicates trunk rotation).

**Table 3:** Characteristics of the stroke patients and health participants.

Characteristic	Patients, N = 30	Healthy Participants, N = 15
Age	67.5 (58.75–72.5)	29 (24.5–44.5)
Female sex (%)	30	60
Left-handed (%)	0	6.7
Days since stroke	64 (33.5–109.25)	-
Diagnosis of hemorrhage (%)	33.3	-
Somatosensory deficits presented (%)	57	-
Brunnstrom recovery stage *, proximal	4 (3–5)	-
Brunnstrom recovery stage *, distal	5 (3–5)	-

Notes: Data are presented as median (interquartile range), unless noted otherwise. IQR, interquatile range. * Brunnstrom recovery stage in the upper extremities; possible range, 1–6.

**Table 4:** Multiple regression analysis with continuous variables of FMA endpoints as objective variables.

FMA Test	Dependent Variable	Coefficient	*p*-Value	R	R2
Flexor synergy	(Intercept)	2.037	0.233	0.907	0.823
	Elbow flexion/extension	0.062	<0.001		
	Shoulder flexion/extension	0.021	0.011		
	Shoulder external/internal rotation	0.052	0.001		
Extensor synergy	(Intercept)	4.024	<0.001	0.857	0.734
	Elbow flexion/extension	−0.079	<0.001		
	Neck rotation	−0.027	0.001		
	shoulder total flexion	0.157	<0.001		
	shoulder flexion/extension	−0.077	0.001		
	shoulder abduction/adduction	0.044	0.014		
Coordination/Speed	(Intercept)	1.848	0.021	0.838	0.721
	Elbow flexion/extension	−0.036	<0.001		
	Shoulder total flexion	0.05	<0.001		

Note: FMA, Fugl–Meyer Assessment.

## Data Availability

The data presented in this study are not available because of reasons concerning the privacy of the participants.

## Supplementary Materials

The following supporting information can be downloaded at: https://www.mdpi.com/article/10.3390/s23115213/s1, Table S1. The correlation coefficient between each joint angle (flexor synergy: 12 points). Table S2. The correlation coefficient between each joint angle (flexor synergy: 10 points). Table S3. The correlation coefficient between each joint angle (flexor synergy: 1 point). Table S4. Discriminant Analysis Summary.
