# Supplementary material for: Attempt to Make the Upper-Limb Item of Objective Fugl–Meyer Assessment Using 9-Axis Motion Sensors
Source: Sensors (Basel). 2023 May 30;23(11):5213. doi: 10.3390/s23115213 (PMC10255665; doi:10.3390/s23115213)
Supplement: Supplementary file 1 [file sensors-23-05213-s001.zip › sensors-2340939-supplementary.pdf]

Table S1. The correlation coefficient between each joint angle (flexor synergy: 12 points).

Pearson's correlation coefficient (N=951)		r≥0.7	r≤0.7													
	Neck flexion/extension	Neck lateral flexion (right/left)	Neck rotation (right/left)	Waist flexion/extension	Waist lateral flexion (right/left)	Waist rotation (right/left)	Chest flexion/extension	Chest lateral flexion (right/left)	Chest rotation (right/left)	Elbow flexion/extension	Shoulder total flexion	Shoulder flexion/extension	Shoulder abduction/adduction	Shoulder external rotation/internal rotation	Palmar flexion/dorsiflexion	Radial flexion/ulnar flexion	Forearm pronation/supination
Neck flexion/extension	1.000	0.832 <0.001	0.626 <0.001	-0.589 <0.001	-0.135 <0.001	-0.196 <0.001	0.405 <0.001	-0.473 <0.001	-0.242 <0.001	-0.641 <0.001	-0.597 <0.001	-0.375 <0.001	-0.610 <0.001	-0.583 <0.001	0.621 <0.001	-0.474 <0.001	-0.555 <0.001
Neck lateral flexion (right/left)	0.832 <0.001	1.000	0.502 <0.001	-0.333 <0.001	-0.327 <0.001	-0.329 <0.001	0.116 <0.001	-0.524 <0.001	-0.077 <0.001	-0.514 <0.001	-0.430 <0.001	-0.439 <0.001	-0.446 <0.001	-0.415 <0.001	0.438 <0.001	-0.149 <0.001	-0.358 <0.001
Neck rotation (right/left)	0.626 <0.001	0.502 <0.001	1.000	-0.871 <0.001	0.253 <0.001	-0.243 <0.001	0.795 <0.001	-0.932 <0.001	-0.786 <0.001	-0.994 <0.001	-0.990 <0.001	-0.051 0.114	-0.992 <0.001	-0.988 <0.001	0.990 <0.001	-0.888 <0.001	-0.960 <0.001
Waist flexion/ extension	-0.589 <0.001	-0.333 <0.001	-0.871 <0.001	1.000	-0.031 0.342	0.401 <0.001	-0.859 <0.001	0.672 <0.001	0.625 <0.001	0.891 <0.001	0.899 <0.001	0.021 0.510	0.898 <0.001	0.907 <0.001	-0.902 <0.001	0.879 <0.001	0.882 <0.001
Waist lateral flexion (right/left)	-0.135 <0.001	-0.327 <0.001	0.253 <0.001	-0.031 0.342	1.000	0.747 <0.001	0.286 <0.001	-0.313 <0.001	-0.542 <0.001	-0.198 <0.001	-0.263 <0.001	0.237 <0.001	-0.258 <0.001	-0.263 <0.001	0.274 <0.001	-0.451 <0.001	-0.315 <0.001
Waist rotation (right/left)	-0.196 <0.001	-0.329 <0.001	-0.243 <0.001	0.401 <0.001	0.747 <0.001	1.000	-0.318 <0.001	0.190 <0.001	0.109 0.001	0.271 <0.001	0.214 <0.001	-0.205 <0.001	0.219 <0.001	0.278 <0.001	-0.201 <0.001	0.009 0.788	0.131 <0.001

Chest flexion/ extension	0.405 <0.001	0.116 <0.001	0.795 <0.001	-0.859 <0.001	0.286 <0.001	-0.318 <0.001	1.000	-0.632 <0.001	-0.869 <0.001	-0.777 <0.001	-0.798 <0.001	0.443 <0.001	-0.800 <0.001	-0.869 <0.001	0.807 <0.001	-0.824 <0.001	-0.777 <0.001
Chest lateral flexion (right/left)	-0.473 <0.001	-0.524 <0.001	-0.932 <0.001	0.672 <0.001	-0.313 <0.001	0.190 <0.001	-0.632 <0.001	1.000	0.760 <0.001	0.913 <0.001	0.895 <0.001	0.037 0.254	0.899 <0.001	0.895 <0.001	-0.888 <0.001	0.728 <0.001	0.852 <0.001
Chest rotation (right/left)	-0.242 <0.001	-0.077 <0.001	-0.786 <0.001	0.625 <0.001	-0.542 <0.001	0.109 0.001	-0.869 <0.001	0.760 <0.001	1.000	0.740 <0.001	0.767 <0.001	-0.518 <0.001	0.767 <0.001	0.831 <0.001	-0.764 <0.001	0.778 <0.001	0.747 <0.001
Elbow flexion/extension	-0.641 <0.001	-0.514 <0.001	-0.994 <0.001	0.891 <0.001	-0.198 <0.001	0.271 <0.001	-0.777 <0.001	0.913 <0.001	0.740 <0.001	1.000	0.993 <0.001	0.112 0.001	0.995 <0.001	0.984 <0.001	-0.991 <0.001	0.883 <0.001	0.967 <0.001
Shoulder total flexion	-0.597 <0.001	-0.430 <0.001	-0.990 <0.001	0.899 <0.001	-0.263 <0.001	0.214 <0.001	-0.798 <0.001	0.895 <0.001	0.767 <0.001	0.993 <0.001	1.000	0.087 0.008	1.000 <0.001	0.986 <0.001	-0.997 <0.001	0.928 <0.001	0.986 <0.001
Shoulder flexion/extension	-0.375 <0.001	-0.439 <0.001	-0.051 0.114	0.021 0.510	0.237 <0.001	-0.205 <0.001	0.443 <0.001	0.037 0.254	-0.518 <0.001	0.112 0.001	0.087 0.008	1.000	0.086 0.008	-0.056 0.087	-0.087 0.007	-0.015 0.646	0.119 <0.001
Shoulder abduction/adduction	-0.610 <0.001	-0.446 <0.001	-0.992 <0.001	0.898 <0.001	-0.258 <0.001	0.219 <0.001	-0.800 <0.001	0.899 <0.001	0.767 <0.001	0.995 <0.001	1.000 <0.001	0.086 0.008	1.000	0.987 <0.001	-0.998 <0.001	0.924 <0.001	0.983 <0.001
Shoulder external rotation/internal rotation	-0.583 <0.001	-0.415 <0.001	-0.988 <0.001	0.907 <0.001	-0.263 <0.001	0.278 <0.001	-0.869 <0.001	0.895 <0.001	0.831 <0.001	0.984 <0.001	0.986 <0.001	-0.056 0.087	0.987 <0.001	1.000	-0.986 <0.001	0.912 <0.001	0.956 <0.001
Palmar flexion/dorsiflexion	0.621 <0.001	0.438 <0.001	0.990 <0.001	-0.902 <0.001	0.274 <0.001	-0.201 <0.001	0.807 <0.001	-0.888 <0.001	-0.764 <0.001	-0.991 <0.001	-0.997 <0.001	-0.087 0.007	-0.998 <0.001	-0.986 <0.001	1.000	-0.932 <0.001	-0.978 <0.001

Radial flexion/ulnar flexion	-0.474 <0.001	-0.149 <0.001	-0.888 <0.001	0.879 <0.001	-0.451 <0.001	0.009 0.788	-0.824 <0.001	0.728 <0.001	0.778 <0.001	0.883 <0.001	0.928 <0.001	-0.015 0.646	0.924 <0.001	0.912 <0.001	-0.932 <0.001	1.000	0.948 <0.001
Forearm pronation/supination	-0.555 <0.001	-0.358 <0.001	-0.960 <0.001	0.882 <0.001	-0.315 <0.001	0.131 <0.001	-0.777 <0.001	0.852 <0.001	0.747 <0.001	0.967 <0.001	0.986 <0.001	0.119 <0.001	0.983 <0.001	0.956 <0.001	-0.978 <0.001	0.948 <0.001	1.000

Table S2. The correlation coefficient between each joint angle (flexor synergy: 10 points).

Pearson's correlation coefficient (N=2359)			r≥0.7	r≤0.7													
	Neck flexion/extension	Neck lateral flexion (right/left)	Neck rotation (right/left)	Waist flexion/extension	Waist lateral flexion (right/left)	Waist rotation (right/left)	Chest flexion/extension	Chest lateral flexion (right/left)	Chest rotation (right/left)	Elbow flexion/extension	Shoulder total flexion	Shoulder flexion/extension	Shoulder abduction/adduction	Shoulder external rotation/internal rotation	Palmar flexion/dorsiflexion	Radial flexion/ulnar flexion	Forearm pronation/supination
Neck flexion/extension	1.000	0.833 <0.001	-0.550 <0.001	-0.647 <0.001	0.299 <0.001	0.780 <0.001	-0.942 <0.001	-0.830 <0.001	0.648 <0.001	0.844 <0.001	0.840 <0.001	-0.038 0.068	0.856 <0.001	0.894 <0.001	-0.776 <0.001	0.345 <0.001	0.241 <0.001
Neck lateral flexion (right/left)	0.833 <0.001	1.000	-0.630 <0.001	-0.570 <0.001	0.047 0.023	0.809 <0.001	-0.910 <0.001	-0.925 <0.001	0.435 <0.001	0.896 <0.001	0.865 <0.001	0.061 <0.001	0.882 <0.001	0.956 <0.001	-9.955 <0.001	0.288 <0.001	0.566 <0.001
Neck rotation (right/left)	-0.550 <0.001	-0.630 <0.001	1.000	-0.037 0.072	0.201 <0.001	-0.222 <0.001	0.490 <0.001	0.807 <0.001	0.107 <0.001	-0.883 <0.001	-0.898 <0.001	-0.748 <0.001	-0.880 <0.001	-0.758 <0.001	0.624 <0.001	-0.404 <0.001	-0.279 <0.001
Waist flexion/extension	-0.647 <0.001	-0.570 <0.001	-0.037 0.072	1.000	-0.608 <0.001	-0.923 <0.001	0.760 <0.001	0.445 <0.001	-0.903 <0.001	-0.371 <0.001	-0.361 <0.001	0.653 <0.001	-0.393 <0.001	-0.538 <0.001	0.468 <0.001	-0.346 <0.001	-0.146 <0.001

Waist lateral flexion (right/left)	0.299<br><0.001	0.047<br>0.023	0.201<br><0.001	-0.608<br><0.001	1.000	0.389<br><0.001	-0.268<br><0.001	-0.038<br>0.067	0.498<br><0.001	0.017<br>0.415	0.013<br>0.518	-0.497<br><0.001	0.028<br>0.172	0.083<br><0.001	-0.035<br>0,092	0.066<br>0.001	-0.111<br><0.001
Waist rotation (right/left)	0.780<br><0.001	0.809<br><0.001	-0.222<br><0.001	-0.923<br><0.001	0.389<br><0.001	1.000	-0.914<br><0.001	-0.678<br><0.001	0.836<br><0.001	0.614<br><0.001	0.598<br><0.001	-0.454<br><0.001	0.627<br><0.001	0.760<br><0.001	-0.727<br><0.001	0.325<br><0.001	0.409<br><0.001
Chest flexion/extension	-0.942<br><0.001	-0.910<br><0.001	0.490<br><0.001	0.760<br><0.001	-0.268<br><0.001	-0.914<br><0.001	1.000	0.848<br><0.001	-0.721<br><0.001	-0.825<br><0.001	-0.813<br><0.001	0.164<br><0.001	-0.834<br><0.001	-0.910<br><0.001	0.834<br><0.001	-0.335<br><0.001	-0.409<br><0.001
Chest lateral flexion (right/left)	-0.830<br><0.001	-0.925<br><0.001	0.807<br><0.001	0.445<br><0.001	-0.038<br>0.067	-0.678<br><0.001	0.848<br><0.001	1.000	-0.301<br><0.001	-0.969<br><0.001	-0.953<br><0.001	-0.274<br><0.001	-0.960<br><0.001	-0.966<br><0.001	0.888<br><0.001	-0.331<br><0.001	-0.481<br><0.001
Chest rotation (right/left)	0.648<br><0.001	0.435<br><0.001	0.107<br><0.001	-0.903<br><0.001	0.498<br><0.001	0.836<br><0.001	-0.721<br><0.001	-0.301<br><0.001	1.000	0.270<br><0.001	0.282<br><0.001	-0.677<br><0.001	0.311<br><0.001	0.432<br><0.001	-0.316<br><0.001	0.329<br><0.001	0.020<br>0.334
Elbow flexion/extension	0.844<br><0.001	0.896<br><0.001	-0.883<br><0.001	-0.371<br><0.001	0.017<br>0.415	0.614<br><0.001	-0.825<br><0.001	-0.969<br><0.001	0.270<br><0.001	1.000	0.996<br><0.001	0.392<br><0.001	0.997<br><0.001	0.969<br><0.001	-0.864<br><0.001	0.421<br><0.001	0.394<br><0.001
Shoulder total flexion	0.840<br><0.001	0.865<br><0.001	-0.898<br><0.001	-0.361<br><0.001	0.013<br>0.518	0.598<br><0.001	-0.813<br><0.001	-0.953<br><0.001	0.282<br><0.001	0.996<br><0.001	1.000	0.413<br><0.001	0.999<br><0.001	0.969<br><0.001	-0.864<br><0.001	0.461<br><0.001	0.354<br><0.001
Shoulder flexion/extension	-0.038<br>0.068	0.061<br><0.001	-0.748<br><0.001	0.653<br><0.001	-0.497<br><0.001	-0.454<br><0.001	0.164<br><0.001	-0.274<br><0.001	-0.677<br><0.001	0.392<br><0.001	0.413<br><0.001	1.000	0.378<br><0.001	0.187<br><0.001	-0.108<br><0.001	0.168<br><0.001	0.005<br>0.800
Shoulder abduction/adduction	0.856<br><0.001	0.882<br><0.001	-0.880<br><0.001	-0.393<br><0.001	0.028<br>0.172	0.627<br><0.001	-0.834<br><0.001	-0.960<br><0.001	0.311<br><0.001	0.997<br><0.001	0.999<br><0.001	0.378<br><0.001	1.000	0.967<br><0.001	-0.844<br><0.001	0.453<br><0.001	0.365<br><0.001
Shoulder external	0.894<br><0.001	0.956<br><0.001	-0.758<br><0.001	-0.538<br><0.001	0.083<br><0.001	0.760<br><0.001	-0.910<br><0.001	-0.966<br><0.001	0.432<br><0.001	0.969<br><0.001	0.969<br><0.001	0.187<br><0.001	0.967<br><0.001	1.000	-0.923<br><0.001	0.390	0.425<br><0.001

rotation/internal rotation																<0.001	
Palmar flexion/dorsiflexion	-0.776 <0.001	-9.955 <0.001	0.624 <0.001	0.468 <0.001	-0.035 0,092	-0.727 <0.001	0.834 <0.001	0.888 <0.001	-0.316 <0.001	-0.864 <0.001	-0.864 <0.001	-0.108 <0.001	-0.844 <0.001	-0.923 <0.001	1.000	-0.087 <0.001	-0.671 <0.001
Radial flexion/ulnar flexion	0.345 <0.001	0.288 <0.001	-0.404 <0.001	-0.346 <0.001	0.066 0.001	0.325 <0.001	-0.335 <0.001	-0.331 <0.001	0.329 <0.001	0.421 <0.001	0.461 <0.001	0.168 <0.001	0.453 <0.001	0.390 <0.001	-0.087 <0.001	1.000	-0.431 <0.001
Forearm pronation/supination	0.241 <0.001	0.566 <0.001	-0.279 <0.001	-0.146 <0.001	-0.111 <0.001	0.409 <0.001	-0.409 <0.001	-0.481 <0.001	0.020 0.334	0.394 <0.001	0.354 <0.001	0.005 0.800	0.365 <0.001	0.425 <0.001	-0.671 <0.001	-0.431 <0.001	1.000

Table S3. The correlation coefficient between each joint angle (flexor synergy: 1 point).

Pearson's correlation coefficient(N=1781)	r≥0.7	r≤0.7															
	Neck flexion/extension	Neck lateral flexion (right/left)	Neck rotation (right/left)	Waist flexion/extension	Waist lateral flexion (right/left)	Waist rotation (right/left)	Chest flexion/extension	Chest lateral flexion (right/left)	Chest rotation (right/left)	Elbow flexion/extension	Shoulder total flexion	Shoulder flexion/extension	Shoulder abduction/adduction	Shoulder external rotation /internal rotation	Palmar flexion/dorsiflexion	Radial flexion/ulnar flexion	Forearm pronation/supination
Neck flexion/extension	1.000	-0.633 <0.001	0.182 <0.001	-0.066 0.006	-0.076 0.001	-0.321 <0.001	0.521 <0.001	-0.633 <0.001	-0.580 <0.001	-0.019 0.412	-0.88 <0.001	0.581 <0.001	-0.503 <0.001	-0.470 <0.001	0.360 <0.001	-0.114 <0.001	0.038 0.105
Neck lateral flexion (right/left)	-0.633 <0.001	1.000	-0.058 0.015	-0.152 <0.001	0.117 <0.001	0.073 0.002	-0.302 <0.001	0.368 <0.001	0.521 <0.001	0.231 <0.001	-0.201	-0.329 <0.001	0.270 <0.001	0.313 <0.001	-0.192 <0.001	0.223 <0.001	0.094 <0.001

											<0.001						
Neck rotation (right/left)	0.182<br><0.001	-0.058<br>0.015	1.000	-0.137<br><0.001	-0.518<br><0.001	-0.744<br><0.001	0.638<br><0.001	-0.297<br><0.001	-0.310<br><0.001	-0.025<br>0.291	-0.568<br><0.001	0.373<br><0.001	-0.533<br><0.001	-0.153<br><0.001	0.614<br><0.001	0.372<br><0.001	0.806<br><0.001
Waist flexion/extension	-0.066<br>0.006	-0.152<br><0.001	-0.137<br><0.001	1.000	-0.668<br><0.001	0.139<br><0.001	0.293<br><0.001	0.191<br><0.001	-0.314<br><0.001	-0.769<br><0.001	-0.076<br>0.001	0.314<br><0.001	-0.237<br><0.001	0.252<br><0.001	-0.267<br><0.001	-0.749<br><0.001	-0.184<br><0.001
Waist lateral flexion (right/left)	-0.076<br>0.001	0.117<br><0.001	-0.518<br><0.001	-0.668<br><0.001	1.000	0.507<br><0.001	-0.719<br><0.001	0.148<br><0.001	0.568<br><0.001	0.609<br><0.001	0.451<br><0.001	-0.557<br><0.001	0.616<br><0.001	-0.084<br><0.001	-0.168<br><0.001	0.364<br><0.001	-0.376<br><0.001
Waist rotation (right/left)	-0.321<br><0.001	0.073<br>0.002	-0.744<br><0.001	0.139<br><0.001	0.507<br><0.001	1.000	-0.835<br><0.001	0.725<br><0.001	0.692<br><0.001	0.028<br>0.239	0.668<br><0.001	-0.526<br><0.001	0.807<br><0.001	0.455<br><0.001	-0.699<br><0.001	-0.252<br><0.001	-0.731<br><0.001
Chest flexion/extension	0.521<br><0.001	-0.302<br><0.001	0.638<br><0.001	0.293<br><0.001	-0.719<br><0.001	-0.835<br><0.001	1.000	-0.681<br><0.001	-0.847<br><0.001	-0.412<br><0.001	-0.615<br><0.001	0.801<br><0.001	-0.925<br><0.001	-0.469<br><0.001	0.628<br><0.001	-0.123<br><0.001	0.582<br><0.001
Chest lateral flexion (right/left)	-0.633<br><0.001	0.368<br><0.001	-0.297<br><0.001	0.191<br><0.001	0.148<br><0.001	0.725<br><0.001	-0.681<br><0.001	1.000	0.811<br><0.001	-0.125<br><0.001	0.528<br><0.001	-0.563<br><0.001	0.822<br><0.001	0.655<br><0.001	-0.532<br><0.001	0.023<br>0.342	-0.265<br><0.001
Chest rotation (right/left)	-0.580<br><0.001	0.521<br><0.001	-0.310<br><0.001	-0.314<br><0.001	0.568<br><0.001	0.692<br><0.001	-0.847<br><0.001	0.811<br><0.001	1.000	0.397<br><0.001	0.441<br><0.001	-0.689<br><0.001	0.870<br><0.001	0.552<br><0.001	-0.483<br><0.001	0.326<br><0.001	-0.280<br><0.001
Elbow flexion/extension	-0.019<br>0.412	0.231<br><0.001	-0.025<br>0.291	-0.769<br><0.001	0.609<br><0.001	0.028<br>0.239	-0.412<br><0.001	-0.125<br><0.001	0.397<br><0.001	1.000	-0.052<br>0.028	-0.429<br><0.001	0.247<br><0.001	0.230<br><0.001	-0.186<br><0.001	0.393<br><0.001	-0.161<br><0.001
Shoulder total flexion	-0.88<br><0.001	-0.201	-0.568	-0.076<br>0.001	0.451<br><0.001	0.668<br><0.001	-0.615<br><0.001	0.528<br><0.001	0.441<br><0.001	-0.052<br>0.028	1.000	-0.246<br><0.001	0.754<br><0.001	0.138<br><0.001	-0.403<br><0.001	0.078<br>0.001	-0.505<br><0.001

		<0.001	<0.001														
Shoulder flexion/extension	0.581 <0.001	-0.329 <0.001	0.373 <0.001	0.314 <0.001	-0.557 <0.001	-0.526 <0.001	0.801 <0.001	-0.563 <0.001	-0.689 <0.001	-0.429 <0.001	-0.246 <0.001	1.000	-0.737 <0.001	-0.449 <0.001	0.397 <0.001	-0.176 <0.001	0.308 <0.001
Shoulder abduction/adduction	-0.503 <0.001	0.270 <0.001	-0.533 <0.001	-0.237 <0.001	0.616 <0.001	0.807 <0.001	-0.925 <0.001	0.822 <0.001	0.870 <0.001	0.247 <0.001	0.754 <0.001	-0.737 <0.001	1.000	0.457 <0.001	-0.544 <0.001	0.205 <0.001	-0.470 <0.001
Shoulder external rotation/internal rotation	-0.470 <0.001	0.313 <0.001	-0.153 <0.001	0.252 <0.001	-0.084 <0.001	0.455 <0.001	-0.469 <0.001	0.655 <0.001	0.552 <0.001	0.230 <0.001	0.138 <0.001	-0.449 <0.001	0.457 <0.001	1.000	-0.770 <0.001	-0.261 <0.001	-0.380 <0.001
Palmar flexion/dorsiflexion	0.360 <0.001	-0.192 <0.001	0.614 <0.001	-0.267 <0.001	-0.168 <0.001	-0.699 <0.001	0.628 <0.001	-0.532 <0.001	-0.483 <0.001	-0.186 <0.001	-0.403 <0.001	0.397 <0.001	-0.544 <0.001	-0.770 <0.001	1.000	0.507 <0.001	0.828 <0.001
Radial flexion/ulnar flexion	-0.114 <0.001	0.223 <0.001	0.372 <0.001	-0.749 <0.001	0.364 <0.001	-0.252 <0.001	-0.123 <0.001	0.023 0.342	0.326 <0.001	0.393 <0.001	0.078 0.001	-0.176 <0.001	0.205 <0.001	-0.261 <0.001	0.507 <0.001	1.000	0.583 <0.001
Forearm pronation/supination	0.038 0.105	0.094 <0.001	0.806 <0.001	-0.184 <0.001	-0.376 <0.001	-0.731 <0.001	0.582 <0.001	-0.265 <0.001	-0.280 <0.001	-0.161 <0.001	-0.505 <0.001	0.308 <0.001	-0.470 <0.001	-0.380 <0.001	0.828 <0.001	0.583 <0.001	1.000

Table S4. Discriminant Analysis Summary.

Target test symbol	Selected Variables	FMA score		
		0	1	2
T1	Constant	-1.264	-10.092	-18.867
	Elbow joint flexion/extension	0.076	0.214	0.293
T2	Constant	-3.445	-24.427	-33.182
	Elbow joint flexion/extension	0.149	0.361	0.447
	Chest rotation (left/right)	0.057	0.389	0.327

	Chest lateral flexion (left/right)	-0.141	-0.571	-0.605
T3	Constant	-20.453	-35.048	-41.096
	Shoulder joint total flexion	-0.052	-0.079	0.114
	Shoulder joint internal/external rotation	-0.333	-0.406	-0.249
	Elbow joint flexion/extension	0.478	0.613	0.616
	Waist lateral flexion (left/right)	0.428	1.322	-0.084
	Chest rotation (left/right)	0.079	0.287	0.024
T4	Constant	-7.835	-14.041	-24.920
	Shoulder joint total flexion	0.205	0.456	0.591
	Shoulder joint internal/external rotation	-0.252	0.054	0.194
	Shoulder joint flexion/extension	-0.062	0.090	0.131
	Chest flexion/extension	-0.107	-0.377	-0.447
T5	Constant	-8.996	-44.259	-55.223
	Elbow joint flexion/extension	0.296	0.797	0.918
	Shoulder joint internal/external rotation	-0.317	-0.607	-0.452
	Chest lateral flexion (left/right)	0.029	-0.588	-0.801
	Shoulder joint flexion/extension	-0.067	-0.129	-0.057
T6	Constant	-1.962	-5.405	-11.265
	Shoulder joint total flexion	0.065	0.216	0.338
	Waist rotation (left/right)	0.506	-0.087	-0.355
	Wrist radial flexion/ulnar flexion	-0.085	0.068	0.041
T7	Constant	-4.434	-2.500	-0.122
	Elbow joint flexion/extension	0.131	0.098	0.023
	Wrist radial flexion/ulnar flexion	-0.219	-0.168	-0.009
T8	Constant	-6.972	-9.933	-8.495
	Elbow joint flexion/extension	0.246	0.104	-0.057
	Neck rotation (left/right)	0.148	0.125	0.013
	Shoulder joint total flexion	-0.039	0.432	0.765
	Shoulder joint flexion/extension	0.125	-0.31	-0.293
	Forearm pronation/supination	-0.015	0.056	0.014
T9	Constant	-7.069	-8.051	-7.665
	Elbow joint flexion/extension	0.224	0.3147	-0.032
	Neck rotation (left/right)	0.138	0.132	0.021
	Shoulder joint total flexion	0.036	0.332	0.671
	Shoulder joint flexion/extension	0.113	-0.047	-0.272
T10	Constant	-19.950	-5.982	-12.973
	Forearm pronation/supination	0.200	-0.036	-0.082
	Chest rotation (left/right)	0.550	0.028	-0.180

	Shoulder joint internal/external rotation	0.196	-0.129	-0.210
	Wrist palmar flexion/dorsiflexion	0.151	-0.067	-0.023
	Elbow joint flexion/extension	0.396	0.025	0.058
	Shoulder joint abduction/adduction	-0.152	0.026	-0.098
	Constant	-8.70	-3.707	-10.565
T11	Shoulder joint flexion/extension	0.043	0.138	0.290
	Elbow joint flexion/extension	0.290	0.102	-0.015
	Constant	-11.036	-6.110	-4.860
T12	Shoulder joint internal/external rotation	-0.347	-0.256	-0.237
	Elbow joint flexion/extension	0.122	0.093	0.071
	Constant	-7.904	-5.947	-25.146
	Shoulder joint total flexion	0.032	0..284	0.669
T13	Elbow joint flexion/extension	0.292	0.026	-0.231
	Neck rotation (left/right)	-0.025	-0.029	-0.129
	Constant	-7.516	-16.687	-29.396
	Shoulder joint total flexion	0.215	0.326	0.443
T14	Waist rotation (left/right)	-0.381	-0.307	-0.964
	Chest lateral flexion (left/right)	-0.234	-0.217	0.302
	Neck lateral flexion (left/right)	0.032	0.105	0.344
	Constant	-8.251	-11.162	-4.894
T15	Shoulder joint internal/external rotation	-0.340	-0.409	-0.269
	Forearm pronation/supination	-0.006	-0.073	-0.021
	Constant	-27.053	-54.226	-58.129
	Wrist palmar flexion/dorsiflexion	-0.065	0.013	0.172
T16	Forearm pronation/supination	-0.394	-0.588	-0.553
	Elbow joint flexion/extension	0.596	0.812	0.825
	Chest flexion/extension	0.083	0.163	0.385
	Constant	-2.158	-1.765	-0.963
	Neck flexion/extension	0.165	0.170	-0.005
T17	Wrist palmar flexion/dorsiflexion	-0.095	-0.027	0.049
	Shoulder joint total flexion	0.038	0.048	0.214
	Constant	-0.321	-2.214	-10.287
T18	Wrist palmar flexion/dorsiflexion	0.024	0.089	0.225
	Shoulder joint abduction/adduction	0.097	0.209	0.354
	Constant	-3.232	-3.718	-0.400
T19	Neck flexion/extension	0.203	0.241	0.060
	Chest lateral flexion (left/right)	-0.711	-0.652	-0.289
T20	Constant	-1.677	-0.221	-0.125

	Wrist palmar flexion/dorsiflexion	-0.096	-0.006	0.027
	Shoulder joint flexion/extension	0.203	0.082	0.022
T21	Constant	-2.859	-10.344	-7.694
	Shoulder joint total flexion	0.085	0.221	0.211
	Elbow joint flexion/extension	0.065	-0.006	0.001
	Chest lateral flexion (left/right)	-0.220	-0.635	-0.371
T22	Constant	-3.445	-6.645	-7.224
	Shoulder joint total flexion	0.062	0.160	0.208
	Chest lateral flexion (left/right)	-0.322	-0.410	-0.061
	Elbow joint flexion/extension	0.082	0.026	-0.009
T23	Constant	-3.011	-12.134	-10.387
	Shoulder joint total flexion	0.082	0.291	0.300
	Waist lateral flexion (left/right)	-0.013	0.970	0.546
	Elbow joint flexion/extension	0.067	-0.011	-0.028
	Chest lateral flexion (left/right)	-0.272	-0.559	-0.253
